# HGCS: an online tool for prioritizing disease-causing gene variants by biological distance

**DOI:** 10.1186/1471-2164-15-256

**Published:** 2014-04-03

**Authors:** Yuval Itan, Mark Mazel, Benjamin Mazel, Avinash Abhyankar, Patrick Nitschke, Lluis Quintana-Murci, Stephanie Boisson-Dupuis, Bertrand Boisson, Laurent Abel, Shen-Ying Zhang, Jean-Laurent Casanova

**Affiliations:** 1 St. Giles Laboratory of Human Genetics of Infectious Diseases, Rockefeller Branch, The Rockefeller University, New York, NY, USA; 2 New York Genome Center, New York, NY, USA; 3 Platforme Bioinformatique, Université Paris Descartes, Paris, France; 4 Unit of Human Evolutionary Genetics, Institut Pasteur, Paris, France; 5 Centre Nationale de la Recherche Scientifique, CNRS URA 3012, Paris, France; 6 Laboratory of Human Genetics of Infectious Diseases, Necker Branch, Inserm UMR 1163, Paris, France; 7 Paris Descartes University, Imagine Institute, Paris, France; 8 Pediatric Immunology-Hematology Unit, Necker Hospital for Sick Children, Paris, France; 9 Howard Hughes Medical Institute, New York, NY, USA

## Abstract

**Background:**

Identifying the genotypes underlying human disease phenotypes is a fundamental step in human genetics and medicine. High-throughput genomic technologies provide thousands of genetic variants per individual. The causal genes of a specific phenotype are usually expected to be functionally close to each other. According to this hypothesis, candidate genes are picked from high-throughput data on the basis of their biological proximity to core genes — genes already known to be responsible for the phenotype. There is currently no effective gene-centric online interface for this purpose.

**Results:**

We describe here the human gene connectome server (HGCS), a powerful, easy-to-use interactive online tool enabling researchers to prioritize any list of genes according to their biological proximity to core genes associated with the phenotype of interest. We also make available an updated and extended version for all human gene-specific connectomes. The HGCS is freely available to noncommercial users from: http://hgc.rockefeller.edu/.

**Conclusions:**

The HGCS should help investigators from diverse fields to identify new disease-causing candidate genes more effectively, via a user-friendly online interface.

## Background

The identification of causal links between human genotypes and disease phenotypes is a key challenge in human genomics, genetics and medicine. The high-throughput data generated by next-generation sequencing (NGS), microarray studies, genome-wide association studies (GWAS) and copy number variation (CNV) provide thousands of variants per individual [1-6]. Most bioinformatic methods for identifying genes potentially associated with specific phenotypes [7-9] are not optimized for Mendelian traits with complete or incomplete clinical penetrance, because they lack the metrics for estimating the relatedness of genes not belonging to the same biological function pathway, or because they generate complex networks that are difficult to interpret, resulting in low discovery rates for disease-causing alleles in high-throughput studies [10].

The causal genes of a specific phenotype are generally expected to be functionally close to each other [11-13]. Candidate genes are therefore picked from high-throughput data on the basis of their biological proximity to core genes — genes already known to be responsible for the phenotype. We recently developed a novel approach, the “human gene connectome” (HGC). The HGC consists of a method and database describing the set of *in silico*-predicted biologically plausible routes and distances between all pairs of human genes. We used this method to generate a “gene-specific connectome” for each human gene, making it possible to rank all human genes in terms of their biological proximity to a core gene of interest. We have demonstrated that the HGC is an effective approach for identifying Mendelian disease-causing genes in high-throughput genetic data, by the ranking of genes according to their biological proximity to core genes known to be associated with the phenotype of interest, as demonstrated by a case study of herpes simplex encephalitis (HSE) and TLR3 pathway genes [10].

We present here the human gene connectome server (HGCS): a novel, effective and easy-to-use interactive online interface through which users can submit any gene list generated by high-throughput techniques (or specific candidate genes of interest) for automatic ranking in terms of biological distance and connectivity *p*-value to the known core genes of the phenotype of interest, and the predicted route between the genes of interest. The HGCS is based on the HGC-derived concept of biological distance between gene pairs (that are either directly or indirectly connected), and provides, for the first time, an opportunity for investigators of all backgrounds to prioritize independently lists of genes of any size, according to their biological distance to core genes. We also provide a new database of 14,129 human gene-specific connectomes. We demonstrate the power of the HGCS for prioritizing candidate genes, with whole-exome sequencing (WES) data from 16 patients with HSE [14], Mendelian susceptibility to mycobacterial disease (MSMD) [15], or invasive pneumococcal disease (IPD) [16]. We compare HGCS with state-of-the-art methods.

## Implementation

### Generation of the HGC and of all human gene-specific connectomes

We extracted data for all direct human protein-protein physical interactions from the updated String version 9.05 (328,391 direct protein-protein binding interactions in the current version, versus 146,566 in the previous version, and a higher level of accuracy) [9] and inverted the interaction confidence scores to obtain direct biological distances, which we used to create a weighted graph of all available human genes. We applied a shortest distance algorithm to find the biological distance and route between all pairs of human genes, to generate the full HGC, with the Python NetworkX package for complex network analysis [17]. We then generated a gene-specific connectome, by ranking all human genes according to their HGC-predicted biological distance to a core gene. We repeated the process for all human genes (See Itan, *et al., 2013* for a comprehensive description of the methodology). The human gene-specific connectomes are available for use and can be downloaded from: http://lab.rockefeller.edu/casanova/HGC.

### Database and online server implementation

The full HGC and gene annotation data are stored on a server, as indexed tables in a MySQL database. All human gene-specific connectomes were converted into a MySQL table. The gene aliases and annotations were compiled from Ensembl BioMart [18,19]. The main code for ranking and annotations was written in PHP, so that it could be run directly from the server. The program uses mysqli_query() commands to access the database and generate queries. The program is designed to maximize gene discovery, by automatically detecting gene aliases if the input is not the conventional gene name, and adding the full gene name (e.g. Toll-like receptor 3 for *TLR3*) and alternative aliases.

### Computing resources and programming languages

We generated the new HGC and all derived human gene-specific connectomes with a Mac Pro computer with a 12-core Intel CPU and 96 GB RAM. The initial data filtering and text mining of the String database were performed with the Perl programming language. The HGC, gene-specific connectomes and simulations were generated with the Python programming language. The HGCS is hosted on The Rockefeller University LAMP shared server, with a VMware instance of 4 GB.

## Results

### The human gene connectome server (HGCS)

**Figure 1:**
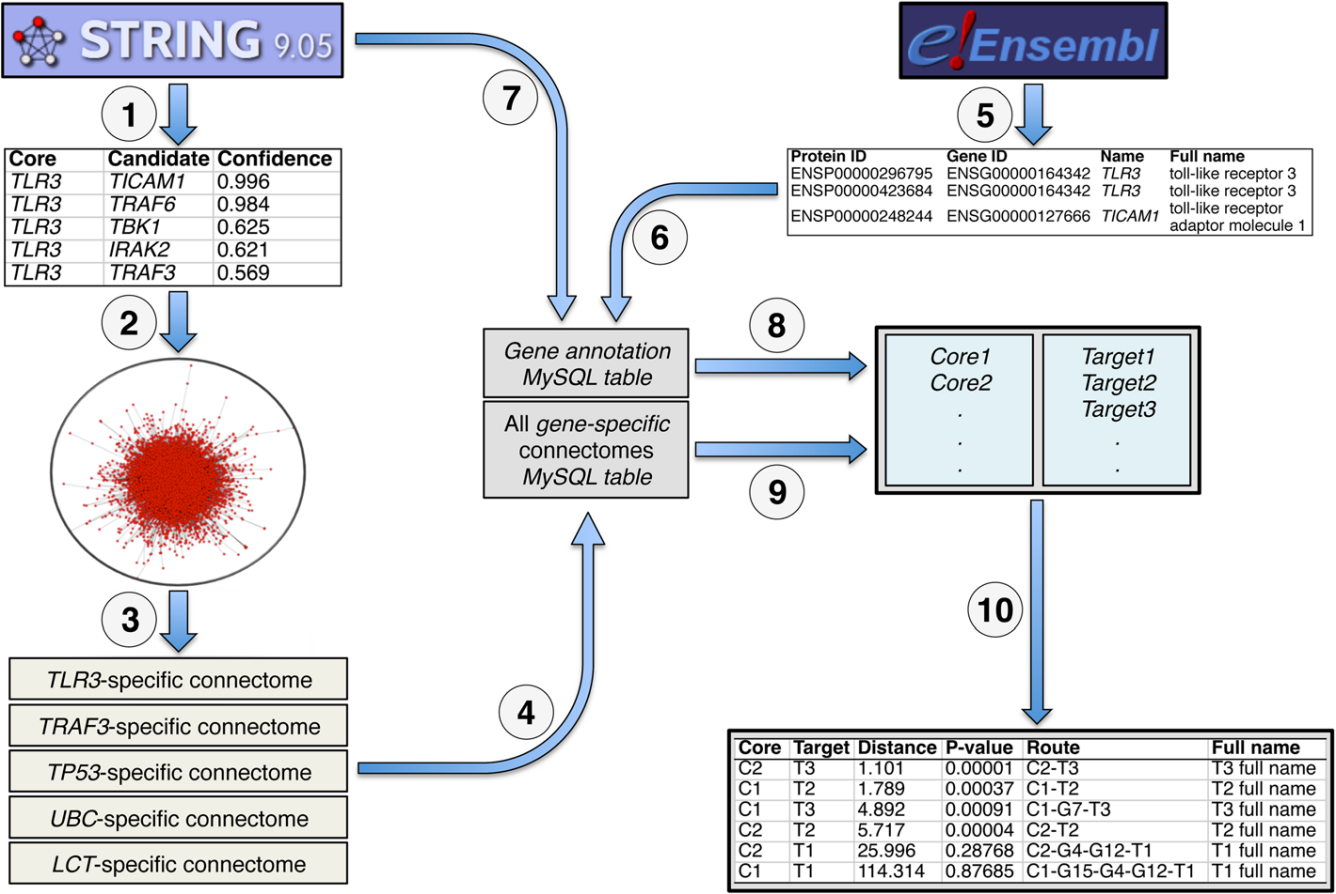
**Schematic representation of the generation, data structure and workflow of the HGCS. (1)** Extraction of all human direct protein-protein binding interactions and the corresponding confidence scores from String. **(2)** Inversion of confidence scores to give direct biological distance metrics and generation of a genome-wide human weighted network. **(3)** Generation, for each human gene, of a *gene*-specific connectome — the set of all other human genes ranked according to their biological proximity to the specific gene. **(4)** Generation of a MySQL table from all human *gene*-specific connectomes. **(5)** Extraction, from Ensembl BioMart, of all human protein IDs, gene IDs, and their corresponding conventional and full names. **(6,7)** Generation of a MySQL table of all alternative gene names for each human gene. **(8,9)** Establishment of the full set of query gene names by identifying missing genes with alternative gene name aliases, extracting the target genes from the connectomes of the core genes. **(10)** Sorting of the target genes according to user-defined metrics, by relatedness to any of the core genes, or separated by core gene. The screen output can then be downloaded as a tab-separated text file.

**Figure 2:**
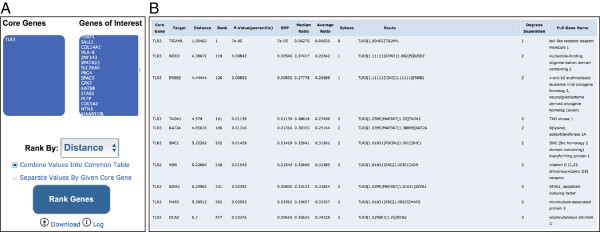
**The HGCS interface. (A)** The two boxes contain the list of genes to prioritize/analyze (which can be acquired from any high-throughput experiment after the application of filters; alternatively, any user-defined list of candidate genes can be used), and the core genes (known to be associated with the phenotype) for ranking purposes. A scroll box allows a choice of metrics for ranking (distance, *p*-value, or best reciprocal *p*-value), and the user may choose whether to rank the results globally, or separately by core gene. **(B)** The output consists of a table of genes ranked with respect to the core genes, which can be downloaded as a tab-separated text file. The information about the nature of connectivity between the core and target genes provided includes HGC-predicted biological distance, ranking of the target gene in the connectome of the core gene, the ratio between biological distance and the genome-wide median and mean biological distances to the core gene, the sphere of the target gene around the core gene, degrees of separation between the genes, and the full gene name.

The HGCS is a gene prioritization and connectivity online interface based on biological distance, which allows users to generate queries about any set of core and target genes. This system can be used for the rapid prediction of the biological distance and connection route between any two given genes of interest, and for the effective prioritization of any number of genes generated by high-throughput methods, on the basis of their biological distance to core genes associated with the human trait of interest or, alternatively, on the basis of *p*-value or best reciprocal *p*-value (BRP, the smallest of the mutual *p*-values between the core and target genes accounting for central and isolated genes). A schematic representation of the HGCS generation workflow is shown in Figure 1. The output can be sorted by proximity to any of the core genes provided, or internally separated by core gene. Figure 2 shows screenshots of the HGCS online platform, demonstrating the ranking of 284 genes from WES data for an HSE patient, using *TLR3* as the core gene. The true HSE-causing gene for this patient, *TICAM1* (*TRIF*), was ranked #1 among the 284 genes. The human gene connectome server is available from: http://lab.rockefeller.edu/casanova/HGC.

### Assessment of the performance of the HGCS

We assessed the power of the HGCS to detect Mendelian disease-causing mutations from the WES data of 16 patients with severe Mendelian diseases: 7 patients with HSE, 7 patients with MSMD and 2 patients with IPD. The genes with disease-causing mutations in the HSE patients were shown experimentally to be *TICAM1* (*TRIF*, in two patients), *TRAF3*, *TBK1* (in two patients), and *UNC93B1* (in two patients) [20-23]. The genes with disease-causing mutations in the MSMD patients were shown experimentally to be *IFNGR2* (in two patients), *ISG15*, *STAT1*, *IL12RB1* (in two patients each), and *IL12B*[24-28]. *RBCK1* was identified as the gene with disease-causing mutations in the IPD patients [16]. We performed standard filtering for the variants: (i) excluding synonymous variations, (ii) keeping rare variations, with a frequency <1% in the 1000 Genomes [29] and NHLBI Exome Variant Server (http://evs.gs.washington.edu/EVS/) databases, and (iii) accounting for sequencing batch effects and highly mutated genes (which are less likely to be morbid) by in-house filtering of variants appearing in more than 0.6% of the patients in all disease cohorts other than for the specific disease tested (0.6% being the most stringent criterion that does not filter out the true disease-causing gene in all patients, for which filtering allows the removal of false-positive genes abundant in WES data because they are naturally highly mutated or due to sequencing errors causing the same false mutations to appear in various WES samples).

Filtering decreased the median number of variant genes per patient to 301. We chose *TLR3* as the core gene for HSE, *IFNG* as the core gene for MSMD, and *IKBKG* as the core gene for IPD, because these genes have been experimentally validated as central genes in the gene pathways associated with the pathogenesis of these diseases [14-16]. We used the HGCS to rank all gene variants for each patient according to biological proximity to the core gene associated with the patient’s disease. We then compared the performance, interface and functions of the HGCS with those of two other state-of-the-art methods: (i) FunCoup, using the MaxLink interface, which ranks top interactors, and (ii) HumanNet, which ranks by top subnetworks [7,8]. In both FunCoup and HumanNet, we added the relevant core genes to the analyses, and chose the first cluster/subnetwork containing the true disease gene.

### The human gene-specific connectome database

We generated and made available 14,129 human gene-specific connectomes, each containing the set of all human genes ranked by their biological proximity to the specific core gene of interest. Each gene-specific connectome contains the following data categories regarding the nature of the connection between the core gene and the target genes: HGC-predicted biological distance, rank among all human genes according to distance to the core gene, *p*-value for connectivity, BRP, the ratio between the core gene—target gene distance and the median distance between the core gene and all human genes, the ratio between the core gene—target gene distance and mean distance between the core gene and all human genes, the sphere around the core gene (simplified percentile metrics), the predicted route (i.e. the genes between the core and target genes), degrees of separation (the number of direct connections between the core and target genes), and the full name of the target gene. All human gene-specific connectomes are available from: http://lab.rockefeller.edu/casanova/HGC.

### Comparison of the HGCS with state-of-the-art methods

We assessed the ability of the HGCS to prioritize candidate genes in high-throughput data, using WES data for 16 patients who suffered from herpes simplex encephalitis (HSE, core gene *TLR3*, Additional files 1, 2, 3, 4, 5, 6 and 7: Table S1-S7) [14], Mendelian susceptibility to mycobacterial disease (MSMD, core gene *IFNG*, Additional files 8, 9, 10, 11, 12, 13 and 14: Table S8-S14) [15], or invasive pneumococcal disease (IPD, core gene *IKBKG*, Additional files 15 and 16: Tables S15 and S16) [16] due to single-gene inborn errors of immunity. There was a median of 301 WES-filtered genes per patient. Additional files 1, 2, 3, 4, 5, 6 and 7: Tables S1-S7 show the prioritized WES genes for each HSE patient, together with the connectivity between these genes and *TLR3* predicted by the HGCS. Additional files 8, 9, 10, 11, 12, 13 and 14: Tables S8-S14 show the prioritized WES genes for each MSMD patient, and Additional files 15 and 16: Tables S15 and S16 show the prioritized WES genes for each IPD patient. The true HSE-causing genes (*TICAM1* in two patients, *TBK1* in two patients, *UNC93B1* in two patients and *TRAF3* in a single patient) were ranked #1 in all seven patients, in terms of biological proximity to *TLR3* among the WES-filtered genes, *P* = 4.148E^-17^. The true MSMD-causing genes (*IFNGR2* in two patients, *IL12RB1* in two patients, *ISG15, STAT1*, and IL12B in single patients) were ranked #1 in five patients and #2 in two patients, in terms of biological proximity to *IFNG* among the WES-filtered genes, *P* = 1.243E^-16^. The true IPD-causing gene (*RBCK1* in two patients) was ranked #15 in one patient and #18 in the second patient, in terms of biological proximity to *IKBKG* among the WES-filtered genes, *P* = 0.00185.

We compared the results obtained with those for two other state-of-the-art methods (summarized in Additional files 17: Table S17): (i) FunCoup: the true disease gene was ranked 3 of 29, 7 of 29 and 1 of 29, in 3 of the 16 patients (for the detection of *TICAM1* in HSE and *ISG15* and *STAT1* in MSMD, respectively; the true disease-causing gene was not ranked in the remaining nine patients); (ii) HumanNet (allowing the analysis of a maximum of 250 genes at a time, rather than being core gene-centered): the true disease-causing gene was ranked between #5 and #38 of 43 to 137 clusters in 12 patients, and was not ranked in the remaining four patients. FunCoup and HumanNet cannot rank genes relative to a core gene, and the prediction therefore relates to a significant subnetwork containing the true disease-causing gene. Predictions also involve manual browsing of the output, making these methods less feasible for situations in which ranking on the basis of several core genes is desired.

The HGCS differs from the FunCoup and HumanNet interfaces in several major ways. FunCoup is based on direct interactors or highly connected networks, and is therefore particularly powerful for predicting closely related genes. By contrast, HumanNet was designed for the discovery of new genes in a pathway, and is therefore more suitable for more distantly related genes. HumanNet provided results for 12 of the 16 patients (versus only 3 patients for FunCoup and all 16 patients for the HGCS). Neither FunCoup nor HumanNet is gene-centric. These methods are therefore unable to rank a list of genes according to their biological proximity to a set of genes of interest, and they provide no information about the route connecting human genes of interest. Additional files 17: Table S17 shows comparisons of the performances of the HGCS, FunCoup and HumanNet interfaces for the detection of disease-causing genes from WES data. In summary, for the 16 Mendelian disease-causing genes for the patients whose WES data were studied here, the HGCS outperformed FunCoup in 15 of the 16 tests, and outperformed HumanNet in 14 of the 16 tests.

One of the major aims in studies of Mendelian diseases is to identify, at the single-patient level, a single gene associated with the disease. In this respect, the HGCS is more effective than FunCoup and HumanNet, because it is the only interface that ranks all candidate genes on the basis of their relationship to the given core gene. The other interfaces involve a binary yes/no indication of relatedness to core genes, making it difficult to differentiate between the genes related to the core gene and to identify the specific disease-causing gene. FunCoup and HumanNet are conceptually easier to apply in polygenic/complex genetic studies, as the input for these two interfaces is the full set of candidate genes and there is no need to supply a core gene, and they provide subnetworks that can be inferred to be related to the disease.

## Discussion and conclusions

We present here the HGCS — the first online platform for prioritizing any number of genes on the basis of their biological distance to any number of core genes and the relationships between them. We are making available an updated database of all human gene-specific connectomes. We demonstrate the high performance of the HGCS for high-throughput Mendelian and monogenic studies. We propose an effective method for the use of the HGCS to detect new disease-related genes, based on the collation of central core genes known to be associated with the disease and their use to rank the candidate genes by distance, *P*-value, or BRP (a less stringent scoring, better reflecting the mutual connection when the target gene is less central, but probably associated with a higher false-positive rate). We suggest that *P*-values or BRP could be used to rank lists of gene candidates, rather than for drawing statistical/translational conclusions that a gene is relevant to the phenotype on the basis of statistical significance.

The HGCS performance is dependent upon a reliable selection of core gene(s) associated with the phenotype. This task is straightforward when certain causal genes have already been identified in previous studies. However, in the absence of experimentally validated core genes, the identification of candidate core genes is not trivial. In such cases, we suggest using core genes of the phenotypes most similar to the phenotype of interest, or alternatively using other state-of-the-art approaches described in this work, such as FunCoup and HumanNet. The centrality/connectivity of the selected core genes also influences the HGCS performance, which, in the case of IPD, was decreased with *IKBKG* as a core gene (although still highly significant). We suggest that since *IKBKG* is a highly central/connected gene with a high number of strongly associated genes, it is less effective for differentiating the highly ranked gene candidates. In such cases we propose ranking by additional core genes, if available.

The HGCS has several unique features not found in other state-of-the-art methodologies, including the prediction of meaningful indirect interactions, the provision of a biological distance and route between any two given human genes of interest, and its gene-centric nature, making it particularly useful in diseases or pathways for which associated genes have already been detected and for which the task is detecting and describing new disease- or pathway-associated genes. We anticipate that the rigorous use of the HGCS and the novel concept of biological distance will significantly increase the rate of discovery of new genotype-phenotype causal relationships.

## Availability and requirements

**Project name:** the human gene connectome server (HGCS)

**Project home page:** http://hgc.rockefeller.edu/

**Operating system(s):** platform independent.

**Programming languages:** Python, MySQL, PHP.

**License:** free to noncommercial users.

## Competing interests

The authors declare that they have no competing interests.

## Authors’ contributions

YI conceived, organized and supervised the project, generated the gene-specific connectomes, and conducted the performance and statistical tests. MM and BM planned, designed, and implemented the online server interface. AA, PN and LQM contributed to the data analyses. SBD and BB provided whole exome sequencing data from patients and expertise with candidate genes prioritization. LA, SYZ and JLC assisted in project planning, implementation, and large-scale interpretations of the results.

## Supplementary Material

Additional file 1: Table S1 HGCS-prioritized genes for the first HSE patient for whom *TICAM1* (*TRIF*) was the experimentally validated disease-causing gene. This table shows all filtered gene variants identified by WES for the patient, ranked according to their HGC-predicted biological proximity to *TLR3*. The true HSE-causing gene, *TICAM1* (*TRIF*), in this patient was ranked 1^st^. Click here for file

Additional file 2: Table S2 HGCS-prioritized genes for the second HSE patient for whom *TICAM1* (*TRIF*) was the experimentally validated disease-causing gene. This table shows all filtered gene variants identified by WES for the patient, ranked according to their HGC-predicted biological proximity to *TLR3*. The true HSE-causing gene, *TICAM1* (*TRIF*), in this patient was ranked 1^st^. Click here for file

Additional file 3: Table S3 HGCS-prioritized genes for the first HSE patient for whom *TBK1* was the experimentally validated disease-causing gene. This table shows all filtered gene variants identified by WES for the patient, ranked according to HGC-predicted biological proximity to *TLR3*. The true HSE-causing gene, *TBK1,* was ranked 1^st^. Click here for file

Additional file 4: Table S4 HGCS-prioritized genes for the second HSE patient for whom *TBK1* was the experimentally validated disease-causing gene. This table shows all filtered gene variants identified by WES for the patient, ranked according to their HGC-predicted biological proximity to *TLR3*. The true HSE-causing gene, *TBK1,* was ranked 1^st^. Click here for file

Additional file 5: Table S5 HGCS-prioritized genes from an HSE patient for whom *TRAF3* was the experimentally validated disease-causing gene. This table shows all filtered gene variants identified by WES for the patient, ranked according to their HGC-predicted biological proximity to *TLR3*. The true HSE-causing gene, *TRAF3,* was ranked 1^st^. Click here for file

Additional file 6: Table S6 HGCS-prioritized genes for the first HSE patient for whom *UNC93B1* was the experimentally validated disease-causing gene. This table shows all filtered gene variants identified by WES for the patient, ranked according to HGC-predicted biological proximity to *TLR3*. The true HSE-causing gene, *UNC93B1,* was ranked 1^st^. Click here for file

Additional file 7: Table S7 HGCS-prioritized genes for the second HSE patient for whom *UNC93B1* was the experimentally validated disease-causing gene. This table shows all filtered gene variants identified by WES for the patient, ranked according to HGC-predicted biological proximity to *TLR3*. The true HSE-causing gene, *UNC93B1,* was ranked 1^st^. Click here for file

Additional file 8: Table S8 HGCS-prioritized genes from the first MSMD patient for whom *IFNGR2* was the experimentally validated disease-causing gene. This table shows all filtered gene variants identified by WES for the patient, ranked according to their HGC-predicted biological proximity to *IFNG*. The true MSMD-causing gene, *IFNGR2,* was ranked 1^st^. Click here for file

Additional file 9: Table S9 HGCS-prioritized genes from the second MSMD patient for whom *IFNGR2* was the experimentally validated disease-causing gene. This table shows all filtered gene variants identified by WES for the patient, ranked according to their HGC-predicted biological proximity to *IFNG*. The true MSMD-causing gene, *IFNGR2,* was ranked 1^st^. Click here for file

Additional file 10: Table S10 HGCS-prioritized genes from an MSMD patient for whom *ISG15* was the experimentally validated disease-causing gene. This table shows all filtered gene variants identified by WES for the patient, ranked according to their HGC-predicted biological proximity to *IFNG*. The true MSMD-causing gene, *ISG15,* was ranked 2^nd^. Click here for file

Additional file 11: Table S11 HGCS-prioritized genes from an MSMD patient for whom *STAT1* was the experimentally validated disease-causing gene. This table shows all filtered gene variants identified by WES for the patient, ranked according to their HGC-predicted biological proximity to *IFNG*. The true MSMD-causing gene, *STAT1,* was ranked 1^st^. Click here for file

Additional file 12: Table S12 HGCS-prioritized genes from the first MSMD patient for whom *IL12RB1* was the experimentally validated disease-causing gene. This table shows all filtered gene variants identified by WES for the patient, ranked according to their HGC-predicted biological proximity to *IFNG*. The true MSMD-causing gene, *IL12RB1,* was ranked 1^st^. Click here for file

Additional file 13: Table S13 HGCS-prioritized genes from the second MSMD patient for whom *IL12RB1* was the experimentally validated disease-causing gene. This table shows all filtered gene variants identified by WES for the patient, ranked according to their HGC-predicted biological proximity to *IFNG*. The true MSMD-causing gene, *IL12RB1,* was ranked 2^nd^. Click here for file

Additional file 14: Table S14 HGCS-prioritized genes from an MSMD patient for whom *IL12B* was the experimentally validated disease-causing gene. This table shows all filtered gene variants identified by WES for the patient, ranked according to their HGC-predicted biological proximity to *IFNG*. The true MSMD-causing gene, *IL12B,* was ranked 1^st^. Click here for file

Additional file 15: Table S15 HGCS-prioritized genes from the first IPD patient for whom *RBCK1* was the experimentally validated disease-causing gene. This table shows all filtered gene variants identified by WES for the patient, ranked according to their HGC-predicted biological proximity to *IKBKG*. The true IPD-causing gene, *RBCK1,* was ranked 15^th^. Click here for file

Additional file 16: Table S16 HGCS-prioritized genes from the second IPD patient for whom *RBCK1* was the experimentally validated disease-causing gene. This table shows all filtered gene variants identified by WES for the patient, ranked according to their HGC-predicted biological proximity to *IKBKG*. The true IPD-causing gene, *RBCK1,* was ranked 18^th^. Click here for file

Additional file 17: Table S17 Comparison of the performances of the HGCS and other state-of-the-art methods for the detection of disease genes in WES data. This table shows rankings obtained with the HGCS, HumanNet and FunCoup (for a median of 301 genes per patient) for the true HSE, MSMD and IPD disease-causing genes in the exomes of 16 patients. Click here for file
