# Supplementary material for: HGCS: an online tool for prioritizing disease-causing gene variants by biological distance
Source: BMC Genomics. 2014 Apr 3;15:256. doi: 10.1186/1471-2164-15-256 (PMC4051124; doi:10.1186/1471-2164-15-256)
Supplement: Additional file 17: Table S17 — Comparison of the performances of the HGCS and other state-of-the-art methods for the detection of disease genes in WES data. This table shows rankings obtained with the HGCS, HumanNet and FunCoup (for a median of 301 genes per patient) for the true HSE, MSMD and IPD disease-causing genes in the exomes of 16 patients. [file 1471-2164-15-256-S17.DOCX]

| **Disease Causing Gene** | **Disease Core Gene** | **Disease** | **Rank in HGCS** | **Rank in HumanNet** | **Rank in FunCoup** |
| --- | --- | --- | --- | --- | --- |
| *TICAM1* #1 | *TLR3* | HSE | 1/216 | 16/78 | NA |
| *TICAM1* #2 | *TLR3* | HSE | 1/266 | 7/70 | 3/29 |
| *TBK1* #1 | *TLR3* | HSE | 1/193 | 17/77 | NA |
| *TBK1* #2 | *TLR3* | HSE | 1/187 | 6/76 | NA |
| *TRAF3* | *TLR3* | HSE | 1/235 | 5/85 | NA |
| *UNC93B1* #1 | *TLR3* | HSE | 1/191 | NA | NA |
| *UNC93B1* #2 | *TLR3* | HSE | 1/259 | NA | NA |
| *IFNGR2* #1 | *IFNG* | MSMD | 1/216 | 12/77 | NA |
| *IFNGR2* #2 | *IFNG* | MSMD | 1/260 | 11/43 | NA |
| *ISG15* | *IFNG* | MSMD | 2/243 | 38/68 | 7/29 |
| *STAT1* | *IFNG* | MSMD | 1/194 | 5/69 | 1/29 |
| *IL12RB1* #1 | *IFNG* | MSMD | 1/182 | 22/65 | NA |
| *IL12RB1* #2 | *IFNG* | MSMD | 2/263 | NA | NA |
| *IL12B* | *IFNG* | MSMD | 1/254 | NA | NA |
| *RBCK1* #1 | *IKBKG* | IPD | 15/394 | 6/137 | NA |
| *RBCK1* #2 | *IKBKG* | IPD | 18/370 | 11/121 | NA |
